# Exploring the impact of high reliability organization implementation on patient handling injury rates: a cross-sectional study

**DOI:** 10.1186/s12995-025-00487-0

**Published:** 2025-11-19

**Authors:** Michael Joshua G. Morales, Pauline Hilton, OiSaeng Hong, Stella Bialous, Marie Martin, Mary Foley, Soo-Jeong Lee

**Affiliations:** 1 University of California, San Francisco, San Francisco, CA USA; 2 Department of Veterans Affairs, Washington, DC USA

**Keywords:** High reliability organization, Organizational culture, Patient handling injury, Health care staff, Safe patient handling and mobility, Musculoskeletal disorders

## Abstract

**Background:**

Patient handling injuries (PHIs) are among the leading causes of workplace injuries among healthcare workers. High-reliability organization (HRO) programs aim to prevent detrimental errors and improve organizational safety. This study examined the relationship between HRO implementation and direct care staffs’ PHI rates.

**Methods:**

This cross-sectional study used data from 124 Veterans Health Administration (VHA) facilities. HRO implementation was measured by program duration and HRO climate. Negative binomial regression models were used to examine the relationship between HRO implementation and PHI rates, progressively adjusting for staff-to-patient ratio, facility type, size, and complexity.

**Results:**

A longer HRO program duration was associated with higher HRO climate (*p* = .01), but HRO implementation (duration and climate) was not associated with PHI rates. Higher staff-to-patient ratio was associated with lower PHI rates (*p* < .05). Ambulatory care service facilities had lower PHI rates than acute care facilities (β = -0.824, *p* = .03). The most clinically complex facilities (level 1a) had higher PHI rates than the level 3 least complex facilities (β = 0.806, *p* = .04).

**Conclusion:**

While this study did not observe a significant relationship between HRO implementation and PHI rate among direct care staff, the study findings highlight the importance of adequate staffing for injury prevention and the need to consider facility type and complexity in PHI prevention efforts. Future research is needed to explore the impact of HRO on worker safety, using more sophisticated measures to assess HRO programs and climate.

## Introduction

Musculoskeletal disorders (MSDs) are highly prevalent among health care workers worldwide, which are largely associated with ergonomic risks from lifting heavy patients and maintaining awkward postures [1]. According to the U.S. Bureau of Labor Statistics, the incidence rate of MSDs involving lost workdays was 38.4 per 10,000 full-time equivalents (FTE) for the health care industry in 2018, and this rate was substantially higher than the rates for the construction industry (28.9) and all industries in the private sector (27.2) [2]. Health care workers lost a median of 8 days of work due to MSDs [2]. MSDs of health care workers not only affect their quality of life and job satisfaction but also the quality of care that they provide to patients [3–6]. Organizational efforts for worker safety are critically needed to prevent MSDs among health care workers.

High Reliability Organizations (HRO) are organizations that operate in complex and high-risk environments but consistently perform safely, efficiently, and with high quality [7–9]. Workplace safety is in the center of the concept of HRO. The HRO concept first emerged in the aviation, nuclear, and military industries and then has been adopted in health care [10–12]. In 1998, the Institute of Medicine (IOM) introduced the HRO concept in health care, and the Agency for Healthcare Research and Quality endorsed the concept in 2005 to reduce health care-related errors and injuries [10, 13]. 

HROs are guided by five fundamental principles, including “preoccupation with failure,” “reluctance to simplify,” “sensitivity to operations,” “commitment to resilience,” and “deference to expertise” [12, 14, 15]. The principle of *preoccupation with failure* encourages organizations to view near-misses as learning opportunities, enhancing vigilance even in the absence of overt errors. The principle of *reluctance to simplify* emphasizes the complexity of failures and discourages oversimplified explanations. The principle of *sensitivity to operation* calls for heightened awareness of potential threats or errors in systems and processes. The principle of *commitment to resilience* focuses on constant error correction to mitigate any potential organizational impact. The principle of *deference to expertise* give priority to the knowledge and insights of the most informed individuals, regardless of their hierarchical position [14, 15]. HRO principles foster a culture of safety by promoting a “just” culture wherein blaming individuals for errors is discouraged, building staff trust in leadership, learning from the organization’s errors, and adapting to rapidly changing operational situations [12, 16, 17]. 

Research on HRO in health care has examined the impact of HRO practices on patient safety outcomes. Several studies have shown that HRO implementation is associated with improved patient safety environment and reduced rates of patient adverse events such as hospital-acquired infections, medication errors, and patient falls [9, 15, 18, 19]. While evidence supporting the positive impact of HRO practices on patient safety is increasing, there is a paucity of research on staff safety outcomes. One study examined the impact of implementing HRO principles on workplace burnout and found significant improvements in psychological safety and emotional exhaustion and a decrease of staff burnout rates by 52% after implementing an HRO initiative [20]. HRO practices may also positively affect physical safety of workers but the impact of HRO implementation has not yet been examined. Therefore, to address the research gap, the present study examined the relationship between HRO implementation and patient handling injury (PHI) incidence among health care workers in Veterans Health Agency (VHA) facilities.

## Methods

### Study design and setting

The study is a cross-sectional ecological study using data from 124 VHA facilities that provided complete data necessary for the data analysis. The study sample represented 81.6% of 152 VHA facilities in the US. The VHA health care systems include a network of outpatient clinics, residential nursing facilities, veterans’ centers, and acute medical centers [21]. In 2019, the VHA launched an agency-wide implementation of HRO [9, 15]. Beginning in 2019, the VHA facilities were divided into three cohorts for asynchronous HRO implementation. The first cohort, initiated in 2019, had 20 sites; the second cohort, initiated in 2021, had 54 sites; and the last cohort, initiated in 2022, consisted of the remaining 78 sites. Given the cohorts’ different initiation years, their HRO progressions may differ across the cohorts and also HRO practices may differ from facility to facility.

### Data sources

The study obtained de-identified, facility-level data from two VHA program offices: (a) HRO module data from the VHA HRO Office, and (b) Annual Safe Patient Handling and Mobility (SPHM) Survey data from VHA SPHM Office.

### HRO module

The HRO module is a self-reported questionnaire developed by the VHA HRO office to measure the individual staff’s perception on the implementation status of HRO practices. The HRO module was introduced in the All-Employee Survey (AES) in 2024. The AES mandates that all VHA employees complete the survey as part of their annual assessment of their workplace culture, job satisfaction, and leadership visibility.

The five-item HRO module questionnaire was based on the ADKAR model, a change model that was initially used to facilitate individual adoption of changes that support more extensive organizational efforts [22]. Each of the questionnaire’s questions corresponds to a domain identified by the VHA HRO office: alignment, motivation, understanding, empowerment, and leadership. The VHA HRO module’s domain has elements that are equivalent to the ADKAR model’s elements. *Awareness*, equivalent to the VA alignment domain, measures individuals’ understanding of the necessity and possible impact of a change [23]. *Desire*, related to the motivation domain, assesses whether staff are willing to embrace the change and actively participate in its implementation [23]. *Knowledge*, corresponding to the understanding domain, evaluates whether staff have the knowledge and skills necessary to facilitate the change [23]. *Ability*, linked to the empowerment domain, measures whether staff have the resources, skills, and capacity to support and implement the change [23]. Last, *reinforcement* pertains to the leadership domain, which assesses whether staff have the ongoing support and commitment needed to sustain the change [23]. The HRO module data were used to create a HRO climate variable.

### Annual SPHM survey

Every year, the VHA SPHM Central Office surveys all VHA facilities to assess the status of their SPHM programs and ensure compliance with the agency-wide SPHM directive, VHA 1611. The survey is completed by the SPHM program manager from each facility. This study obtained 2024 SPHM survey data for the facility characteristics and PHI incidence that occurred during 2024. PHIs are self-reported by the staff to their local VHA’s Safety Office, which records the injuries through the Occupational Safety and Health Administration Form 300. PHI data are then relayed to the SPHM program manager for surveillance and injury investigation, and the annual aggregate number for the facility is entered into the survey form.

## Study variables

### PHI rate

*PHI*, the dependent variable, was defined as an injury resulting from lifting, handling, repositioning, or assisting patients with mobility problems according to the VHA SPHM office [21]. The PHI definition excludes injuries caused by patient assaults. The PHI incidence rate was calculated for each facility as the number of PHI divided by the total number of direct care staff, which included all licensed and unlicensed personnel involved in patient care (e.g., nurses, imaging and rehabilitation staff, transport personnel, certified nurse’s assistants, and social workers). The PHI rate of the individual VA facility was expressed as “per 1,000 direct care staff.”

### HRO implementation

HRO implementation, an independent variable, was measured by HRO duration and HRO climate.

**HRO Duration** is the length of time since the facility implemented HRO principles. There were three cohorts by implementation year. Cohort 1 facilities began HRO programs in 2019 and had the longest HRO duration. Cohort 2 facilities started HRO in 2021 and Cohort 3, the most recent group, implemented HRO in 2022.

**HRO Climate** was created using the 5-item HRO module questionnaire. It is important to note that the HRO module in the AES was not explicitly designed to measure HRO climate, but we considered that responses to the HRO module items together reflect the environment wherein the extent to which HRO is embedded in the organization. Content validity was assessed through expert review, and it was determined that the five items reasonably capture HRO climate. The internal consistency of the HRO module questionnaire in the study sample was excellent, with a Cronbach’s alpha of 0.93. For construct validity, convergent and divergent validity were evaluated using items from the AES survey’s Patient Safety Culture Survey [24]. The HRO climate showed higher correlations with overall patient safety climate (*r* = .68), risk mitigation (*r* = .73), and just culture (*r* = .66) compared to work group cooperation (*r* = .42) and organizational respect (*r* = .43).

A total of 175,810 direct care staff responded to the HRO module. Due to the mandatory nature of the HRO module within the AES survey each VA facility had HRO module data available for analysis. The response to each question is through a 5-point Likert scale ranging from 1 (strongly disagree) to 5 (strongly agree). The VA HRO office then aggregates the individual responses to calculate the overall mean score for each domain, representing the implementation status of HRO practices of VA facilities.

### Facility characteristics

The facility characteristics included bed capacity, facility type, facility size by number of direct care staff, facility complexity, and staff-to-patient ratio levels. Bed capacity refers to beds licensed to operate in either an acute care hospital or long-term care facility. The SPHM survey asks bed capacity for acute care and long-term care separately using the following categories: for acute care beds > 200 beds (large), 101 to 200 beds (medium), 1 to 100 beds (small), and none; and for long-term care bed, > 100 beds (large), 26 to 100 beds (medium), 1 to 25 beds (small), and none. Facility type was derived from the bed capacity variables and classified into four categories including both acute and long-term care services, mainly acute care, mainly long-term care, or ambulatory care only. As the bed capacity variable used different categories for acute care beds and long-term care beds, we created a facility size variable based on the total number of direct care staff in a VHA facility and categorized it based on tertiles: large (≥ 2,000), medium (1,000–1,999), and small (< 1,000). Facility complexity level reflects the range of services offered, which includes different care and surgery types, teaching and research capabilities, and patient volume. The VHA classifies facilities into five levels of complexity: the 1a (the most complex), 1b, 1c, 2, and 3 (the least complex). Staffing is an important factor affecting patient handling injury risks. Using available data in the surveys, the staff-to-patient ratio variable was created to reflect staffing as the number of direct care staff divided by the total number of unique patients served by each VHA facility. Only staff assigned to direct clinical roles (e.g., nursing, rehabilitation, transport) were included in the numerator. Patient counts as the denominator were based on the total number of unique patients served by each VHA facility. The staff-to-patient ratio was also categorized into four groups based on quartiles: ≤2.0, > 2.0 to 2.7, > 2.7 to 3.8, and > 3.8 direct care staff per 100 patients.

### Data analysis

Data analysis was conducted using Stata version 17. Descriptive statistics, such as frequencies, percentages, means, medians, standard deviations, and minimum and maximum values, were used to summarize the study variables. The power analysis was conducted with a significance level (α) of 0.05 and a desired power of 0.8, involving 124 facilities. Through simulation, the power analysis determined that this study could detect an incidence rate ratio of 0.87 or greater.

Applying the central limit theory, the distributions of the HRO climate (alignment, motivation, understanding, empowerment, and leadership) and PHI rate variables were assumed to approximate normal distributions. The differences in the HRO climate and PHI rates by facility characteristics were examined using either ANOVA for normally distributed variables, or the Kruskal-Wallis test for variables with a skewed distribution.

Given that the PHIs are count data and the variable exhibited overdispersion, negative binomial regression was used to examine the relationship between HRO implementation variables and PHI rates. The outcome variable was modeled as the count of PHI events, with the number of direct care staff included as an offset to account for differences in the population at risk across facilities. Using the number of direct care staff as an offset normalized the PHI rate, allowing for comparisons across facilities while controlling for variations in staff size. Multivariable analysis modeling was conducted to examine the relationships between HRO implementation and PHI rates, by adding facility characteristics as covariates in different models. Bed capacity variables were excluded from the multivariable analysis models to avoid redundancy with facility size by number of direct care staff. Model 1 included HRO duration as the sole predictor, and Model 2 included HRO climate as the sole predictor. Model 3 included both HRO duration and HRO climate, along with staff-to-patient ratio. Model 4 added facility type to Model 3. Model 5 further included facility size and facility complexity, as the most comprehensive model in examining the relationship between HRO implementation and PHI rates. This approach allowed for the identification of significant facility characteristics for PHI rates and the best-fitting model with the optimal balance of simplicity and accuracy was identified by the Bayesian Information Criterion (BIC) [25]. Lower BIC values indicated a better balance between model fit and complexity; therefore, the model with the lowest BIC was selected as the most effective in explaining the data.

## Results

The facility characteristics of the study sample (*N* = 124) are described by the HRO duration cohorts in Table 1. Most facilities provided both acute and long-term care (67.7%), were classified as level 1a (the most clinically complex facilities; 29.8%), and were medium-sized facility based on direct care staffing (42.7% had 1,000 to 1,999 direct care staff). During 2024, the number of veterans seen per facility had a median of 50,047 (IQR: 32,806; 77,963).

### HRO duration and HRO climate

The 124 VHA facilities had a mean HRO climate of 3.78 (*SD* = 0.1). Table 2 presents the HRO climate scores of VHA facilities by the HRO duration cohort. There was a significant difference in the overall HRO climate score across HRO duration cohorts, with a noticeable declining trend by shorter duration (*p* = .01). Facilities that began HRO implementation in 2019 (Cohort 1) have the highest HRO climate score (*M* = 3.83; *SD* = 0.07), followed by those that started in 2021 Cohort (*M* = 3.75; *SD* = 0.09) and 2022 Cohort (*M* = 3.75; *SD* = 0.01).

The scores of each HRO climate domain of alignment, empowerment, and leadership were significantly different by HRO implementation duration (*p* < .05). The 2019 cohort facilities had the highest scores across these domains: alignment (4.01), empowerment (3.70), and leadership (3.72). These scores are followed by 2021 Cohort and 2022 Cohort; however, the scores for the HRO domains of motivation and understanding did not differ significantly across the implementation cohorts.

### HRO implementation and PHI rates

The 124 VHA facilities had a median PHI rate of 3.3 per 1,000 direct care staff (*IQR* = 1.6, 6.1). Table 3 shows the HRO climate scores and PHI rates by facility characteristics, with most facility characteristics showing no significant differences in either outcome. Among facility characteristics, the HRO climate significantly differed only by facility size (*p* = .04) wherein smaller-sized facilities (< 1,000 direct staff) had the highest mean HRO climate of 3.81 (*SD* = 0.11). PHI rates significantly differed only by facility complexity (*p* = .05) wherein level 2 facilities had the highest median PHI rate of 4.44 (*IQR* = 2.14, 7.33).

Table 4 shows the five negative binomial models for the relationships between HRO duration, HRO climate, and PHI rates. In all models, the HRO duration and HRO climate showed no statistically significant association with PHI rates. However, a number of facility characteristics showed a significant relationship with PHI rates. In Model 3 including HRO duration, HRO climate, and staff-to-patient ratio, staff-to-patient ratio was negatively associated with the PHI rate (β = -0.011, 95% CI: -0.021, -0.002). In Model 4 further adding facility type to Model 3, staff-to-patient ratio (β = -0.012, 95% CI: -0.021, -0.002) remained the statistical significance and facility type showed a significant association with PHI rates. Facilities with ambulatory care services only (β = -0.824, 95% CI: -1.576, -0.073) had significantly lower PHI rates compared to facilities only providing acute care services. In Model 5 further including facility size and facility complexity, staff-to-patient ratio and facility type lost their statistical significance while facility complexity emerged as the only significant variable; level 1a facilities with the most complexity had a significantly higher PHI rate compared to the least complex level 3 facilities (β = 0.803, 95% CI: 0.056, 1.551).

## Discussion

The study is the first study that examined the relationship between HRO implementation and the PHI incidence. However, it is disappointing that the study failed to observe a significant association between HRO implementation and PHI rates among the direct care staff in VHA facilities. HRO principles are centered on the workplace safety, but these findings may suggest that the positive impact of HRO implementation in health care focusing on patient safety does not reach staff safety outcomes, such as lowering PHI rates. To compare our findings and explore possible explanations, a review of previous studies on HRO and worker injury in non-healthcare settings was conducted; however, no relevant studies were found. This lack of prior research underscores the novelty of the current study and its contribution as an initial step in building evidence on the potential relationship between HRO implementation and occupational safety outcomes for staff in both health care and non-health care sectors.

A key finding in this study is the enhancement of HRO climate by longer implementation of HRO programs, specifically in the domains of alignment, empowerment, and leadership. The finding suggests that the longer HRO has been implemented, the more ingrained its HRO principles have become in the organizational culture, potentially shaping staff perceptions and leading to a higher HRO climate. This finding is consistent with a systematic review, which found that the staff behaviors and perception toward safety culture programs were more positive in organizations with longer implementation periods [26]. On the other hand, the lack of significant changes in the domain of motivation and understanding over time may imply that these domains remain stable over time, likely due to uniform onboarding and training practices that provide a consistent level of awareness, regardless of how long HRO has been implemented. Leadership must recognize that HRO implementation requires a long-term commitment with results that may not be immediately evident and organizational climate changes take time to evolve. Additionally, this study indicates that HRO climate varies by facility size, with smaller facilities demonstrating a higher HRO climate. A possible explanation is that the implementation and integration of HRO principles may be easier to facilitate in smaller facilities with smaller staff populations. A successful implementation of HRO principles in larger facilities should involve visible leadership commitment, role specific education and training, mechanism for accountability and feedback across the system, and implementation strategies design for a larger staff population [14, 27, 28]. 

While HRO implementation showed no association with PHI rate, higher staff-to-patient ratio was related to lower PHI rate. The findings are consistent with previous studies, wherein inadequate staffing results into assigning more patients per staff member, increases the risk of PHI due to increased demand of patient handling activities, and was associated with higher MSDs and musculoskeletal pain [29, 30]. In addition to PHI, inadequate staffing also increases risks of other occupational injuries such as needlestick injuries [31–33]. 

This study identified that facility type, and facility complexity were associated with PHI rates, although not consistently across all models. VHA facilities with mainly acute care facilities compared to ambulatory care services appeared to have a higher PHI rate, likely due to patients in acute care facilities being more dependent. Increased patient dependency increases the frequency of patient handling tasks, thereby increasing the risk of injury. A study on health care facilities in Minnesota showed that outpatient organizations consistently had the lower PHI rates compared to the inpatient settings [34]. Level 1a or the most complex VHA facilities appeared to have a higher PHI rates in contrast with level 3 or the least complex VHA facilities which are primarily ambulatory services with fewer patient handling activity demands. The higher volume of procedures performed and higher acuity of patients in these facilities can lead to more frequent patient turning and repositioning, further elevating injury risk. The findings suggest that inpatient settings need more tailored efforts to better support direct care staff in performing patient handling tasks and mitigate their higher injury risk.

### Strength and limitation

The study has strengths. First, the mandatory nature of the SPHM program survey and HRO module through the AES resulted in 82% of VHA facilities having complete data for analysis. Second, the study was able to assess HRO implementation across diverse VHA facilities nationwide, allowing for an examination of how facility complexity, staffing size, and duration of implementation influence HRO climate. This variation provides a broad yet system-specific perspective, allowing for a more comprehensive understanding of HRO implementation within the VHA. Moreover, the characteristics of facilities excluded in the study were generally similar to those facilities included in the study, with no notable differences observed.

This study has several limitations. First, the cross-sectional ecological study design limits the ability to infer causal relationships from the study findings. The study designed may not have accounted for possible confounders at the facility level that may contribute to ecological bias. Ecological bias is the incorrect assumption that associations at the facility level also exist at the individual level [35]. To reduce the possible impact of the ecological bias, future studies should adjust for possible confounders that account for contextual differences between facilities such as the facilities’ proportion of obese patients and employee’s skill level. Second, data limitations included the use of a single year of observation and facility-level aggregation. Data from only a single year may not accurately provide reliable outcome data or true variability in PHI rate overtime. The use of facility-level data did not allow the examination of variations at the departmental or unit level, where different areas may have varying degrees of HRO implementation and PHI risk. This aggregation may have obscured the association between HRO duration or climate and PHI incidence rate. Future studies should consider using multi-year data and a more granular, unit level data to enable a more nuanced understanding of the relationship between HRO implementation and PHI rate including the potential for lag effects and considering variations across different units. Third, the staffing-to-patient ratio did not distinguish the direct care staff’s individual professions or specific care areas such as the intensive care unit. Fourth, the HRO climate measure, which used the HRO module questionnaire was originally intended to assess HRO implementation practices. Although construct validity testing supported the adequacy of the HRO climate measure, the strong correlations with patient safety and related constructs suggest potential conceptual overlap. Future research is needed to develop a more nuanced and valid instrument for measuring HRO climate. Lastly, there is possible introductions of response bias such as social desirability bias. Direct care staff may have over-reported their responses to the HRO climate questionnaire to reflect a better adoption of HRO practices. Conversely, underreporting of PHI across facilities may have occurred, potentially distorting the observed associations with the variables.

## Conclusion

This is the first study that explored the impact of HRO implementation on PHIs among direct care staff in VHA facilities. While it did not find a significant association between them, the study provides some helpful insights into this unexplored area in HRO. This study provides a promising evidence that with longer HRO implementation, organizations were more likely to recognize the integration of HRO into their culture. However, the findings should not diminish the value of HRO implementation for promoting a culture of safety in health care. Rather, the relationship between HRO implementation and PHI rate still warrants further investigation using a more sophisticated HRO measurement tool, stratifying injuries by severity, and identifying whether specific mediating factors or implementation strategies contribute to improvements in staff safety. This study highlights the important role of adequate staffing in reducing PHIs. Moreover, PHI prevention strategies should consider facility characteristics and prioritize inpatient units and highly complex facilities where risks may be more pronounced.

**Table 1:** Characteristics of veterans healthcare administration facilities by the high reliability organization (HRO) implementation cohort (*n* = 124)

Variable	Total	HRO Cohort by Implementation Year		
		Cohort 1: 2019 (*n* = 20)	Cohort 2: 2021 (*n* = 49)	Cohort 3: 2022 (*n* = 55)
	*N* (%)	*N* (%)	*N* (%)	*N* (%)
Hospital Setting				
Both Acute and Long-Term	84 (67.7)	16 (80.0)	34 (69.4)	34 (61.8)
Mainly Acute Care	21 (16.9)	2 (10.0)	10 (20.4)	9 (16.4)
Mainly Long-Term Care	12 (9.7)	2 (10.0)	4 (8.2)	6 (10.9)
Ambulatory Care Only	7 (5.7)	0	1 (2.0)	6 (10.9)
Acute Care Beds				
Large (> 200)	13 (10.5)	2 (10.0)	7 (14.3)	4 (7.3)
Medium (101–200)	28 (22.6)	6 (30.0)	12 (24.5)	10 (18.2)
Small (1- 100)	64 (51.6)	10 (50.0)	25 (51.0)	29 (52.7)
None	19 (15.3)	2 (10.0)	5 (10.2)	12 (21.8)
Long-Term Care Beds				
Large (> 100)	37 (29.8)	4 (20.0)	18 (36.7)	15 (27.3)
Medium (26–100)	49 (39.5)	11 (55.0)	16 (32.7)	22 (40.0)
Small (1–25)	10 (8.1)	3 (15.0)	4 (8.2)	3 (5.5)
None	28 (22.6)	2 (10.0)	11 (22.5)	15 (27.3)
Facility Size by the number of direct care staff				
Large (≥ 2,000)	38 (30.7)	7 (35.0)	17 (34.7)	14 (25.5)
Medium (1,000–1,999)	53 (42.7)	7 (35.0)	22 (44.9)	24 (43.6)
Small (< 1000)	33 (26.6)	6 (30.)	10 (20.4)	17 (30.9)
Facility Complexity ^a^				
Level 1a (most complex)	37 (29.8)	7 (35.0)	20 (40.8)	10 (18.2)
1b	21 (16.9)	3 (15.0)	11 (22.5)	7 (12.7)
1c	25 (20.2)	3 (15.0)	6 (12.2)	16 (29.1)
2	13 (10.5)	2 (10.0)	6 (12.2)	5 (9.1)
3 (least complex)	28 (22.3)	5 (25.0)	6 (12.2)	17 (30.9)
Number of Direct Care Staff (Median, IQR)	1,554.5 (936.5; 2,373.5)	1,417.5 (812; 3,409)	1,803 (1,166; 2,809)	1368 (850, 2010)
Number of Unique Veterans Seen (Median, IQR)	50,047 (32,806; 77,963)	50,652.5 (29,949; 82,093)	59,294 (39,267; 95,410)	44,069 (27,335; 60,000)
Staff to Patient Ratio (per 100 patients, Mean, SD)	3.3 (1.6)	3.5 (1.7)	3.3 (1.6)	3.4 (1.6)

^a^ The facility level of complexity determines the VHA facility’s classification based on the types of care and surgeries provided, teaching and research capabilities, and the volume of patients seen

**Table 2:** High reliability organization (HRO) climate by duration among VHA facility (*n* = 124)

Scale/ Items	HRO Duration			
	Cohort 1 (2019) *N* = 20	Cohort 2 (2021) *N* = 49	Cohort 3 (2022) *N* = 55	*P*
	Mean (SD)	Mean (SD)	Mean (SD)	
Overall HRO Climate	3.83 (0.07)	3.78 (0.11)	3.75 (0.09)	**0.01**
I understand how becoming an HRO aligns with VHA’s mission to provide Veterans with exceptional care. (Alignment)	4.01 (0.05)	3.97 (0.08)	3.95 (0.08)	**0.01**
I am motivated to actively participate in my organization’s journey to HRO. (Motivation)	3.88 (0.07)	3.85 (0.11)	3.83 (0.09)	0.14
I have a strong understanding of HRO concepts to recognize what can be changed within my work unit to promote HRO. (Understanding)	3.85 (0.06)	3.82 (0.10)	3.80 (0.08)	0.11
I feel empowered to make changes needed to help my organization become an HRO. (Empowerment)	3.70 (0.09)	3.64 (0.14)	3.61 (0.11)	**0.02**
I believe my executive leadership is actively committed to the advancement of HRO. (Leadership)	3.72 (0.12)	3.63 (0.18)	3.57 (0.17)	**< 0.01**

Differences among groups between HRO Duration were analyzed using the ANOVA test

Likert Scale: (1-Strongly Disagree, 2-Disagree, 3-Neutral, 4- Agree, 5- Strongly Agree)

**Table 3:** High reliability organization (HRO) climate and patient handling injury (PHI) rate by facility characteristics

		HRO Climate		PHI rate (per 1000 direct care staff)		
Variable	Total	Mean (SD)	*P*	Mean (SD)	Median (IQR)	*P*
All Facilities	124	3.78 (0.10)		4.4 (4.0)	3.3 (1.6, 6.1)	
Hospital Setting						0.31
Both Acute and Long-Term Care	84	3.78 (0.10)	0.89	4.55 (3.85)	3.49 (1.67, 6.73)	
Mainly Acute Care	21	3.78 (0.11)		5.15 (5.14)	3.10 (2.53, 6.90)	
Mainly Long-Term care	12	3.77 (0.11)		3.77 (3.03)	4.85 (0.26, 5.92)	
Ambulatory Clinics Only	7	3.81 (0.09)		1.93 (1.83)	1.28 (0.00, 3.60)	
Acute Care Beds			0.95			0.27
Large (> 200)	13	3.79 (0.08)		4.55 (4.56)	2.91 (1.71, 6.07)	
Medium (101–200)	28	3.78 (0.11)		5.15 (3.57)	4.00 (2.36, 7.42)	
Small (1- 100)	64	3.77 (0.11)		4.48 (4.29)	3.17 (1.52, 6.81)	
None	19	3.78 (0.10)		3.09 (2.75)	3.33 (0.00, 5.74)	
Long-Term Care Beds			0.85			0.47
Large (> 100)	37	3.78 (0.10)		4.17 (3.20)	3.24 (1.61, 6.01)	
Medium (26–100)	49	3.77 (0.11)		5.02 (4.32)	4.12 (1.81, 8.09)	
Small (1–25)	10	3.78 (0.09)		2.71 (1.65)	2.71 (1.69, 3.98)	
None	28	3.79 (0.10)		4.34 (4.72)	3.07 (1.22, 4.19)	
Staff to Patient Ratio (per 100 patients)						0.35
**≤**2.0	22	3.79 (0.12)	0.43	6.40 (5.74)	5.52 (2.15, 8.72)	
>2.0-2.7	24	3.78 (0.10)		4.25 (3.43)	3.59 (1.58, 6.96)	
>2.7–3.8	41	3.76 (0.11)		3.86 (3.10)	3.14 (1.59, 5.02)	
>3.8	37	3.79 (0.09)		4.00 (3.68)	2.86 (1.61, 6.15)	
Facility Size by the number of direct care staff						
Large (≥ 2,000)	38	3.78 (0.09)	**0.04**	3.91 (3.61)	2.91 (1.38, 5.74)	0.55
Medium (1,000–1,999)	53	3.75 (0.10)		4.39 (3.21)	3.84 (1.81, 6.15)	
Small (< 1000)	33	3.81 (0.11)		4.84 (5.11)	3.71 (1.00, 7.84)	
Facility Complexity						0.05
Level 1a (most complex)	37	3.77 (0.10)	0.50	5.76 (4.30)	3.74 (2.39, 8.72)	
1b	21	3.78 (0.11)		2.95 (2.14)	2.53 (1.59, 4.60)	
1c	25	3.77 (0.08)		4.49 (4.35)	3.33 (2.15, 5.31)	
2	13	3.82 (0.13)		4.87 (3.26)	4.44 (2.15, 7.33)	
3 (least complex)	28	3.77 (0.10)		3.51 (4.5)	2.63 (0.26, 5.73)	

^a^Facility size is based on the number of direct care staff in a facility

HRO climate is scored from 1- strongly disagree to 5- strongly agree

Differences among groups between Overall HRO climate were analyzed using the ANOVA test

Differences among groups between PHI rate were analyzed using the Kruskal-Wallis test

**Table 4:** Association between patient handling injury rate and high reliability organization (HRO) implementation (Climate and Duration) in veterans healthcare administration facilities (*N* = 124): negative binomial analysis models

	Outcome: Patient Handling Injury Rate					
Variable	Model 1	Model 2	Model 3	Model 4	Model 5	
	β (95% CI)	β (95% CI)	β (95% CI)	β (95% CI)	β (95% CI)	
HRO Duration (ref. Cohort 3- 2022)						
Cohort 1 (2019)	0.159 (-0.193, 0.512)		0.169 (-0.212, 0.551)	0.115 (-0.274, 0.505)	0.073 (− 0.320, 0.465)	
Cohort 2 (2021)	0.173 (-0.179, 0524)		0.181 (-0.173, 0.536)	0.123 (-0.238, 0.484)	0.022 (− 0.313, 0.357)	
HRO Climate		0.691 (-0.967, 2.349)	0.531 (-1.207, 2.268)	0.622 (-1.104, 2.348)	0.549 (− 1.192, 2.290)	
Staff to Patient Ratio (per 100 patients)			**-0.011 (-0.021**,** -0.002)**	**-0.012 (-0.021**,** -0.002)**	−0.004 (− 0.015, 0.006)	
Hospital Setting (ref. Mainly Acute Care)						
Both Acute and Long-Term Care				-0.101 (-0.526, 0.323)	−0.050 (− 0.491, 0.390)	
Mainly Long-Term care				-0.214 (-0.797, 0369)	−0.024 (− 0.751, 0.703)	
Ambulatory care only				**-0.824** **(-1.576**,** -0.073)**	−0.703 (− 1.506, 0.100)	
Facility Size by the number of direct care staff (ref: small)						
Large (≥ 2,000)					−0.593 (− 1.252, 0.065)	
Medium (1,000–1,999)					−0.182 (− 0.648, 0.285)	
Facility Complexity (ref. 3-Least Complex)						
1a (most complex)					**0.803 (0.056**,** 1.551)**	
1b					0.057 (− 0.645, 0.758)	
Variable	Model 1	Model 2	Model 3	Model 4	Model 5	
	β (95% CI)	β (95% CI)	β (95% CI)	β (95% CI)	β (95% CI)	
1c					0.221 (− 0.488, 0.930)	
2					0.337 (− 0.226, 0.900)	
Bayesian Information Criterion (BIC)	775.7	754.6	749.4	760.3	776.2	

## Data Availability

The data that support the findings of this study are available from Department of Veteran’s Affairs High Reliability Organization Office and Safe Patient Handling and Mobility Office but restrictions apply to the availability of these data, which were used under license for the current study, and so are not publicly available.

## Declarations

### Duplicate publication declaration

This manuscript is derived from Chapter 4 of the doctoral dissertation authored by MJGM, available in the university’s dissertation repository. However, it does not constitute dual publication: deposits in institutional repositories and PhD dissertation are not considered prior publication, and this submission presents expanded interpretation, and findings beyond the dissertation. This manuscript has not been published in any other journal and is not under consideration by any other journal.

## Abbreviations

AES: All Employee Survey
ANOVA: Analysis of Variance
BIC: Bayesian Information Criterion
FTE: Full-time Equivalent
HRO: High Reliability Organization
IOM: Institute of Medicine
IQR: Inter-quartile Range
MSD: Musculoskeletal Disorder
PHI: Patient Handling Injuries
SD: Standard Deviation
SPHM: Safe Patient Handling and Mobility
VHA: Veterans’ Health Administration
